# Double-cone ignition scheme for inertial confinement fusion

**DOI:** 10.1098/rsta.2020.0015

**Published:** 2020-10-12

**Authors:** J. Zhang, W. M. Wang, X. H. Yang, D. Wu, Y. Y. Ma, J. L. Jiao, Z. Zhang, F. Y. Wu, X. H. Yuan, Y. T. Li, J. Q. Zhu

**Affiliations:** 1 Beijing National Laboratory for Condensed Matter Physics, Institute of Physics, Chinese Academy of Sciences, Beijing 100190, People's Republic of China; 2 Key Laboratory for Laser Plasmas and School of Physics and Astronomy, Shanghai Jiao Tong University, Shanghai 200240, People's Republic of China; 3 Collaborative Innovation Center of IFSA (CICIFSA), Shanghai Jiao Tong University, Shanghai 200240, People's Republic of China; 4 Department of Physics, Renmin University of China, Beijing 100872, People's Republic of China; 5 Department of Physics, National University of Defense Technology, Changsha 410073, People's Republic of China; 6 Institute for Fusion Theory and Simulation, Department of Physics, Zhejiang University, Hangzhou 310058, People's Republic of China; 7 School of Physics, Peking University, Beijing 100871, People's Republic of China; 8 National Laboratory of High Power lasers and Physics, Shanghai Institute of Optics and Fine Mechanics, Shanghai 201800, People's Republic of China

**Keywords:** inertial confinement fusion, high energy density physics, laser plasma interactions

## Abstract

While major progress has been made in the research of inertial confinement fusion, significant challenges remain in the pursuit of ignition. To tackle the challenges, we propose a double-cone ignition (DCI) scheme, in which two head-on gold cones are used to confine deuterium–tritium (DT) shells imploded by high-power laser pulses. The scheme is composed of four progressive controllable processes: quasi-isentropic compression, acceleration, head-on collision and fast heating of the compressed fuel. The quasi-isentropic compression is performed inside two head-on cones. At the later stage of the compression, the DT shells in the cones are accelerated to forward velocities of hundreds of km s^–1^. The head-on collision of the compressed and accelerated fuels from the cone tips transfer the forward kinetic energy to the thermal energy of the colliding fuel with an increased density. The preheated high-density fuel can keep its status for a period of approximately 200 ps. Within this period, MeV electrons generated by ps heating laser pulses, guided by a ns laser-produced strong magnetic field further heat the fuel efficiently. Our simulations show that the implosion inside the head-on cones can greatly mitigate the energy requirement for compression; the collision can preheat the compressed fuel of approximately 300 g cm^−3^ to a temperature above keV. The fuel can then reach an ignition temperature of greater than 5 keV with magnetically assisted heating of MeV electrons generated by the heating laser pulses. Experimental campaigns to demonstrate the scheme have already begun.

This article is part of a discussion meeting issue ‘Prospects for high gain inertial fusion energy (part 1)’.

## 1. Introduction

In the quest for controlled nuclear fusion energy, the central ignition concept of inertial confinement fusion (ICF) was proposed in the 1970s [1–3]. With the operation of the National Ignition Facility (NIF) in the USA, significant progress has been made in the indirect-drive central ignition ICF scheme [4–6]. NIF can deliver 192 beams of nanosecond (ns) laser pulses at 3*ω* with a total energy of 1.8 MJ. In 2014, NIF experiments demonstrated energy from fusion reactions exceeding that absorbed by the fusion fuel [4]. Further, recent experiments [5] on the NIF showed that the fusion energy was enhanced to 54 kJ, corresponding, however, to a gain factor Q of only about 0.03. Therefore, there is a long way to achieve ignition of the fusion reaction, mainly due to the low energy coupling efficiency of the laser-produced X-rays with the fuel as well as other technological difficulties [7]. On the other hand, the direct-drive central ignition scheme can obtain much higher energy coupling efficiency [8]. However, much higher compression symmetry than the indirect one is needed. This presents new demands in laser beam uniformity technology. To overcome the long-wavelength and short-wavelength non-uniformities, less than 1% of non-uniformities among different laser pulses [9] and highly uniform intensity profiles of each laser pulse [10,11] are required, respectively.

Apart from central ignition schemes, a fast ignition scheme was proposed as one of the most promising alternatives [12]. Different from the central ignition of ICF, the heating process was separated from the compression process in the fast ignition scheme, so that the compression of the fuel could be carried out at a moderate speed, leading to relaxed requirements on symmetry and stability issues. The pre-compressed fuel is then heated by strong beams of megaelectronvolt (MeV)-level electrons generated by picosecond (ps), petawatt (PW) heating laser pulses [13] after hundreds of micrometres transport. Extensive studies have been devoted to this scheme in the last two decades [14,15]. It was found that the large divergence of the generated electrons [14,16,17] significantly reduces the fuel heating efficiency. To mitigate this difficulty, a cone-inserted target was adopted [18] under certain conditions. But most of the following experiments showed the heating efficiencies [18–21] are too low for ignition. With such a low efficiency, up to hundreds of kilojoule (kJ) of the PW heating laser energy is required. This is beyond the capacity of the current PW laser technology. Other important advanced ignition schemes including shock ignition [22,23] and impact ignition [24,25] have also be proposed to overcome the difficulties of the conventional central ignition schemes.

**Figure 1.**
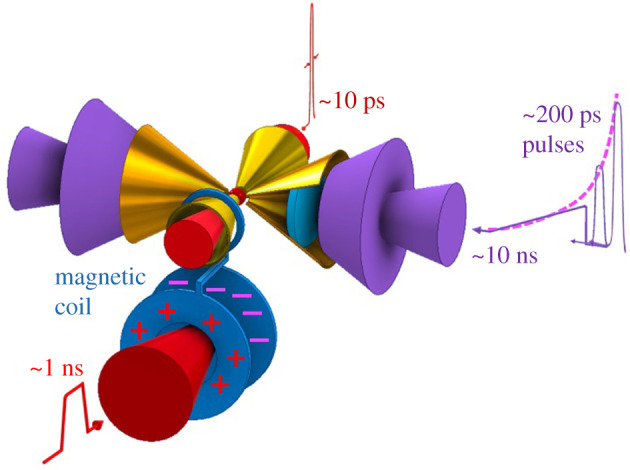
Schematic of the double-cone ignition scheme. The fuel is initially embedded in the entrance of two main cones in the horizontal directions. Both the compression and acceleration processes are implemented in the main cones. Between the cones, there is a vacuum space of approximately 100 µm for the collision process. A couple of additional cones could be placed in the vertical plane to guide the ps, PW heating laser pulses (*ω* or 2*ω*) and the generated hot electron beams. If a magnetically assisted ignition scheme is adopted, only two additional cones along the magnetic field direction are taken. (Online version in colour.)

Here, we propose a double-cone ignition (DCI) scheme (see the schematic in figure 1) to significantly reduce the energy requirement for both the compression and heating laser pulses as well as to enhance the robustness of the compression and heating processes. The DCI scheme includes four separately controllable processes, i.e. quasi-isentropic compression, acceleration, head-on collision and magnetic field-guided fast heating. Spherical compression of the hollow shell of deuterium–tritium (DT) fuels is conducted by quasi-isentropic 10 ns duration laser pulses inside of two head-on gold cones, each as a part of a sphere. Therefore, implosion is performed in the cones in a similar way to that in a sphere, but compression laser pulses with much lower energies are required to form the same fuel density with the same laser power or intensity. At the later stage of the compression, the fuels are accelerated and further compressed by a series of laser pulses with durations of approximately 200 ps via shock waves. This acceleration and compression process make the fuels in the two cones gain forward velocities of hundreds of km s^−1^ as well as high densities of approximately 150 g cm^−3^ when they are ejected from the cone tips. Then, the ejected fuels with high densities collide with each other. Strong Coulomb collisions transfer the forward velocities to thermal ones and the density of the fused fuel is doubled. The preheat of the fuel in the acceleration and collision processes significantly reduces the requirement of the ps heating laser pulses both in energy and power. We expect that the preheated fuel has a temperature up to 1–3 keV and an areal density of greater than 0.8 g cm^−2^. To achieve the fusion energy gain of *Q* > 1, the temperature should be in the range of 5–10 keV. Note that the ps heating pulses can be injected in the plane perpendicular to the axis of the two head-on cones for compression. This presents entire perpendicular space for free set-up in the geometry (see the schematic in figure 1). For example, we can place a number of additional heating cones (say, 2 or more) to guide the ps heating pulses to the fused fuel since these cones do not affect the first three processes occurred in the perpendicular plane. This brings two additional advantages of mitigating the requirement of the heating laser energy as well as enhancing the coupling from the heating pulses to the high-density fuel. Alternatively, we can adopt a laser-produced kT magnetic field [26,27] along two heating pulses to effectively guide the generated MeV electron beams [28]. Our integrated particle-in-cell (PIC) simulations showed that the magnetically assisted approach can significantly enhance the energy coupling from the MeV electrons generated by ps heating laser pulses to the centre of the high-density fuel to a level as high as 14% [28]. The experiments have shown the feasibility of the magnetically assisted approach and enhanced energy coupling [29,30].

A series of simulations and preliminary experiments have been conducted to verify the feasibility and controllability of the four processes of our DCI scheme. In §2, radiation hydrodynamic simulations are presented to form a quasi-isentropic compression with a slow-rising ramp followed by a flat-top laser pulse of moderate-intensity. In §3, our radiation hydrodynamic simulations show that shock waves driven by a series of laser pulses with durations of approximately 200 ps can significantly accelerate the compressed fuel, as well as further enhance the fuel density. Then, in §4, PIC simulations show that the head-on collision of two high-density fuels can greatly increase the fuel density further under some conditions and high-efficiency conversion can be realized from the forward velocities of the two colliding fuels to the thermal energy of the fused fuel. In §5, PIC simulations illustrate that MeV electron beams generated by optimized ps, PW heating laser pulses can heat the fuel to the ignition temperature efficiently, with guiding of a ns laser-produced magnetic field. The Summary and discussion are given in §6 and the laser parameters to be delivered by the proposed upgrading Shenguang II are also presented in table 1.

**Table 1.** The proposed laser parameters of the upgrading Shenguang II facility, which is planned to provide an experimental platform to demonstrate the four processes progressively in next 5 years. The second column shows the ps heating pulses to generate high-energy electron beams and the third one shows the ns pulses to compress the fuels. The pulses for acceleration and magnetic field generation will be taken from the ns pulses and their detailed parameters will be given according to the future optimization based on simulations and experiments.

	ps lasers	ns lasers
beam number	8	32
energy per beam	2.5 kJ/10 ps/1*ω*	8 kJ/10 ns/1*ω* square-pulse
	1.0 kJ/10 ps/2*ω*	5 kJ/10 ns/3*ω* square-pulse
		4 kJ/10 ns/3*ω* triangular-pulse
duration	1–10 ps adjustable	0.2–20 ns adjustable
spot size	3 diffraction limitation (50%)	10 diffraction limitation (95%)
contrast	>10^8^ before 80 ps	≥10^6^
energy fluctuation	≤3% (RMS)	≤3% (RMS)

## 2. Quasi-isentropic compression

In the references of the implosion effects by the ideal isentropic compression laser profile given by Kidder [31,32], we propose a quasi-isentropic compression approach with a slow-rising ramp followed by a flat-top laser pulse of moderate-intensity to conduct a highly efficient compression of DT shells in the gold cones. Because of the moderated and slowly rising laser intensity on targets inside of the cones, the conventional laser–plasma instabilities (LPI) are expected to be controllable. The simulations are carried out by the one-dimensional spherically symmetric radiation hydrodynamic code MULTI-IFE [33]. The target is a cryogenic DT shell with an inner radius of 668 µm. The DT ice has a thickness of 246 µm and a plastic (CH) layer of 30 µm thickness is taken as the ablated material. The initial density of DT ice and CH are 0.25 g cm^−3^ and 1.1 g cm^−3^, respectively. Thus, the mass of the DT ice is 0.49 mg. The target is imploded by focused laser beams of 336 kJ total energy at a wavelength of 0.351 µm.

**Figure 2.**
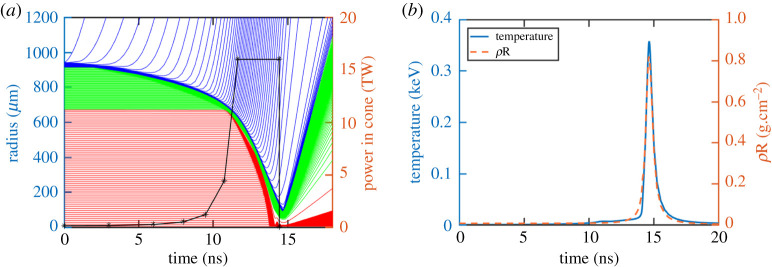
Implosion diagrams (*a*), in which the laser profile on the targets inside of the cones is presented. The evolution of DT ice temperature and areal density (*b*). (Online version in colour.)

The implosion diagram is shown in figure 2*a*, in which the laser pulse profile is also presented. Note that this power only indicates the laser power on target in the cone employed in our experiments and multi-dimensional simulations, where a cone from a solid angle of 2.244 (*Ω *= 2.244 corresponds to a 100° open angle) is taken in the DCI scheme. With a given laser power or intensity for the target compression, the total laser energy required is only *Ω*/4π of that with the spherical implosion. That is, a 2 × 60 kJ laser energy is required in the DCI scheme, instead of 336 kJ used in our one-dimensional spherically symmetric simulations, and the peak power of the laser pulse on the target in each cone is 16 TW. The corresponding mass of the DT ice in each cone is 87 µg. The adiabat keeps low (below 1.3) during the whole implosion process, ensuring an efficient implosion. The maximum implosion velocity reaches 241 km s^−1^ and the peak temperature of the target is 357.6 eV (figure 2*b*). The peak density of the compressed target is 185.6 g cm^−3^, corresponding to a peak areal density of 0.82 g cm^−2^ (figure 2*b*). It is worth noting that, since the impacts of the hydrodynamic instabilities, like Rayleigh–Taylor and Kelvin–Helmholtz instabilities, are not included in our one-dimensional simulations, the peak density could be over-estimated. The multi-dimensional influences will be investigated by multi-dimensional simulations and specially designed experiments in the near future.

Our simulation indicates that the flat-top laser profile is a practical alternative to realize a quasi-isentropic compression, whereas the ideal isentropic compression laser profile usually has a too high peak power for practical applications. To further test the above idea, multi-dimensional radiation hydrodynamic simulations are performed as well as preliminary experiments to demonstrate the quasi-isentropic compression are being carried out at the moment.

We note that the heavy-ion-driven ‘X-target’ [34,35] has a similar slice profile to our ‘double-cone’ target. The ‘X-target’ is designed by removing two cones from opposite sides of an otherwise spherical configuration. Hence, the physical boundaries of both target designs are similar and have nearly the same instabilities; particularly, the Kelvin–Helmholtz instability will arise in both target designs. The multi-dimensional radiation hydrodynamic simulations [34,35] showed via the ‘X-target’ scheme that the maximum density reached is about 120 g cm^−3^, which is close to our peak value of compression before collision obtained by one-dimensional spherical simulation. The multi-dimensional simulations also found that the Kelvin–Helmholtz and Rayleigh–Taylor instabilities have no significant impact on the fuel compression in the ‘X-target’ scheme. Their results on instabilities are valuable for us and we will carefully investigate the instabilities related to our geometry with multi-dimensional simulations and specially designed experiments in the near future.

## 3. Acceleration

**Figure 3.**
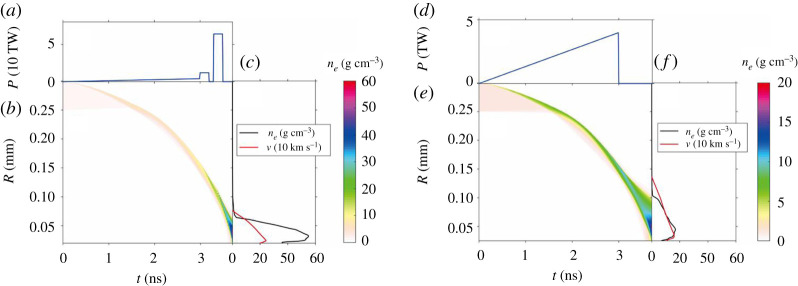
Fuel compression and acceleration by a slope laser pulse, where (*a–c*) corresponds to the case including both the ns compression pulse and two 200 ps acceleration pulses and (*d–f*) corresponds to the case without the acceleration pulses. (*a*) and (*d*) are the laser power profile. (*b*) and (*e*) are the evolution of the fuel density. (*c*) and (*f*) are the fuel density and velocity at the time of 3.7 ns. (Online version in colour.)

Then, we check the acceleration scheme addressed previously. During the later stage of the compression, the fuels in the cones could be accelerated by a series of approximately 200 ps laser pulses via shock waves (see the schematic in figure 1). We run the following two comparative simulations: the first has both the compression ns laser pulse and two 200 ps acceleration laser pulses with higher intensities (see figure 3*a*), while the second does not include the acceleration pulses (figure 3*d*).

We also use the one-dimensional spherically symmetric radiation hydrodynamics code. The compression pulse is a single-slope pulse with an energy of 1.5 kJ, a duration of 3 ns and a peak power of 4 TW, as shown in figure 3*d*. Note that here we do not take a multi-slope pulse to achieve a quasi-isentropic compression. The fuel in the gold cone is replaced by plastic in our simulations. The thickness of the spherical plastic shell is 50 µm and the inner radius of the shell is 250 µm. The tip of the cone is about 40 *µ*m away from the centre of the plastic shell. Our simulation shows that at *t *= 3.7 ns the fuel arrives at the cone tip with a density of 15 g cm^−3^ and a velocity of 150 km s^−1^ (these one-dimensional results are close to our three-dimensional simulation ones given by FLASH code).

A higher velocity of 250 km s^−1^ and a much higher density of 55 g cm^−3^ are achieved when two 200 ps laser pulses are added following the compression ns laser pulse, as shown in figure 3*a–c*. The two 200 ps pulses can drive higher speed shock waves in the compressed fuel, and they can catch up with the one ahead driven by the ns pulse. Finally, the three shock waves driven by the pulses arrive at the cone tip nearly at the same time, which results in high compression efficiency with a high fuel density and speed (figure 3*b*). In our additional simulations, we change the time gaps among the three pulses. It turns out that the three shock waves do not arrive at the radial centre of the shell at the same time. In this case, the fuel density and velocity are decreased significantly. We also find that more acceleration pulses can cause higher velocity and density, but a complex matching among the temporal gaps, pulse intensities and pulse durations is required for optimizations.

The above results show that shock waves driven by a series of approximately 200 ps acceleration pulses can obviously accelerate and further compress the fuel although a low laser energy is taken. With higher energies of acceleration and compression pulses, we expect that this approach would also be valid. In the following work, detailed multi-dimensional simulations with higher pulse energies will be performed to design laser and target parameters for the upcoming acceleration experiments by the end of 2021.

## 4. Head-on collision

In order to investigate the head-on collision of two high-density fuels with forward velocities, we perform a series of two-dimensional PIC simulations using the LAPINS code [36] since the conventional MULTI or FLASH cannot correctly treat the crossing among different fluids. Because high-density plasma interactions are dominated by short-range Coulomb collision in our case, we can ignore the long-range effects. Therefore, we switch off the electromagnetic field solvers in our PIC simulations. In this way, the numerical noise in a simulation of hundreds of ps can also be eliminated. Apart from the classic Coulomb one, our collision model also includes the quantum degenerate effect. We initialize the plasma electrons in the Fermi–Dirac distribution function and the particle scattering is treated via a Pauli blocked binary collision approximation. Such a blocking technique has been widely used in particle transport simulations for semi-conductor devices [37].

**Figure 4.**
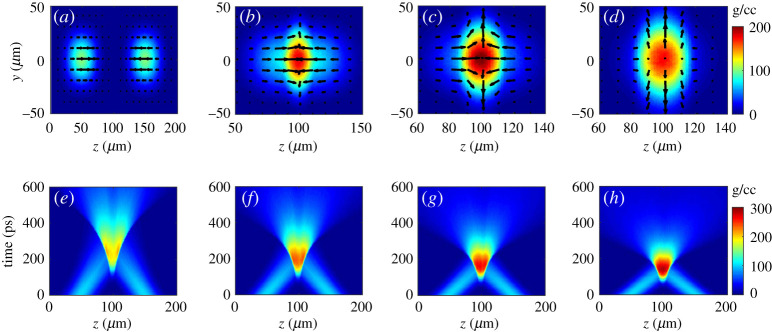
Two-dimensional PIC simulations: the evolution of plasma ion density (*a–d*) at different times of (*a*) 0 ps, (*b*) 165 ps, (*c*) 198 ps and (*d*) 231 ps; the black arrow lines in (*a–d*) indicate the directions of ion currents; the initial density and velocity of the two colliding plasmas are 100 g cm^−3^ and 300 km s^−1^, respectively. One-dimensional PIC simulations: the evolution of plasma ion density with different initial forward velocities of from (*e*) 180 km s^−1^, (*f*) 240 km s^−1^, (*g*) 300 km s^−1^ to (*h*) 360 km s^−1^. The initial plasma density is taken as 100 g cm^−3^. (Online version in colour.)

Figure 4*b,c* shows that the fuel density is doubled to 200 g cm^−3^ via head-on collision of two compressed plasmas with an initial density of 100 g cm^−3^, a forward velocity of 300 km s^−1^ and the temperature of 10 eV. Owing to strong Coulomb collisions, the forward velocities are nearly completely transferred to the fuel thermal energy and the fuel gains a temperature approaching 500 eV. We also simulate the collision of compressed plasmas with different initial parameters: density of 200 g cm^−3^, forward velocity of 300 km s^−1^ and temperature 100 eV. We find that the density doubling and forward velocity transferring to thermal temperature are quite robust. To realize the fully preheat and density doubling, the initial density and velocity of the fuel should be matched. We first scan the initial velocity from 180 km s^−1^ to 360 km s^−1^ with a given density of 100 g cm^−3^. The results are presented in figure 4*e–h*. It is shown that the velocity should be higher than 240 km s^−1^ to realize the fuel density doubled. We also scan the initial density from 50 g cm^−3^ to 150 g cm^−3^ and the results show the initial density should be higher than 100 g cm^−3^. Figure 4*e–h* also show this high-density plasma status can sustain for about 100 ps. We find that the quantumly revised collision model gives a significantly different result from the classic ones. This needs to be further benchmarked. Experiments including both the acceleration and head-on collision processes are planned to implement by the end of 2021.

## 5. Fast heating

Via the head-on collision process, the fused fuel can have a density of 200–300 g cm^−3^ and a temperature approximately 1 keV. This high-density fuel status can sustain approximately 100 ps. During this period, MeV electrons generated by ps heating laser pulses are injected and further heat the fuel to an ignition temperature of 5–10 keV. In our previous work [28], we studied the coupling from the ps heating pulses to the fuel with a density of 300 g cm^−3^ at the centre in a large range of laser intensities. With optimized intensities, the coupling from a 6 ps heating pulses to the high-density core region of the fuel is about 14% with the guidance of an external magnetic field, compared with about 6% via the cone-inserted scheme and 2% without the cone and the external magnetic field. The low coupling in the last two cases is due to the large divergence of the MeV electrons generated by the heating pulses. The external magnetic field at a kT level indeed overcomes the large divergence and well guides the MeV electrons to the core region of the fuel.

**Figure 5.**
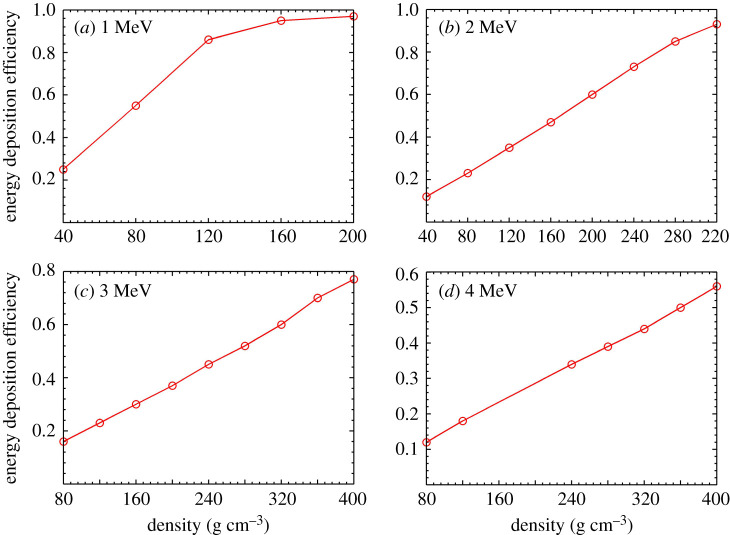
Energy deposition efficiency of hot electrons versus the fuel density with different initial electron energies, where the fuel has a radius of 30 µm of uniform density and the electron beam with a radius of 15 µm and monoenergetic spectrum initially. These results are given by two-dimensional collisional PIC simulations. (Online version in colour.)

The average energy of the hot electrons depends upon the laser intensity and wavelength if the plasma density profile is given. In [38], we found that the electron energy is proportional to $\sqrt{I\lambda^{2}}$ at laser intensity above 10^18 ^W cm^−2^. Using this scaling, one can optimize the laser intensity based on the required electron energy to heat the fuel with a given density. Figure 5 shows our two-dimensional collisional PIC simulations with KLAPS code [39] calculating the electron deposition efficiency to a fuel of a radius of 30 µm. It is shown that a higher density fuel can absorb higher energy electrons more effectively. Note that the 2 MeV electron beam deposits 80% energy to the fuel of 260 g cm^−3^. To obtain this electron energy, the corresponding laser intensity should be about 4 × 10^19^ W cm^−2^ with a wavelength of 0.5 µm, i.e. a 2*ω* ps heating laser. An external magnetic field with a strength lower than 2 kT should guide an electron beam of 2 MeV well according to our previous simulation in [28]. In our design, a ns laser-driven capacitor-coil is applied to generate the magnetic field up to kT level, and the magnetic field could remain its strength for a few ns time scale (the magnetic field life period depends on the driving laser pulse duration), which is longer than the period required for the acceleration, head-on-collision and fast heating processes. In this way, we propose to apply such a magnetic field at (or before) the end of the compression, when the temperature of the fuel plasma is relatively low, partly because the fuel is compressed isentropically. At this time, the fuel plasma is still inside the compression cones and the magnetic field, originated from the coil, needs to efficiently diffuse through the cone and plasma with an estimated time around 1 ns. The delay time between the laser pulse generating the magnetic field and the acceleration laser pulse should be adjusted based on this diffusion time, to allow the external magnetic field to diffuse into the fuel plasma.

To ignite a fuel with a density of 260 g cm^−3^, a radius of 30 µm and an areal density about 0.8 g cm^−2^, the temperature in the fuel should be heated to above 5 keV. This corresponds to inner energy of 5 kJ. Assuming that the fuel could be preheated to 2 keV via the acceleration and collision processes, the ps heating laser pulse (*ω* or 2*ω*) is required to have an energy of 21 kJ if we adopt the energy coupling of 14% from the heating pulse to the fuel core with the magnetically assisted scheme. This is the on-target energy of the upgrading plan for ps heating laser pulses of the Shenguang II laser facility.

## 6. Summary and discussion

In summary, we have proposed the DCI scheme to mitigate the energy requirement for both the compression and heating laser pulses and to overcome some technological difficulties appearing in the conventional ICF central ignition schemes. In the DCI scheme, the complicated central ignition implosion process is replaced by four separately controllable processes: quasi-isentropic compression, acceleration, collision and fast heating. The compression is implemented in two head-on cones from a part of a sphere to significantly reduce the requirement for the compression energy from the ns laser pulses. Through the acceleration and collision, the fuel is significantly preheated to provide 20–30% inner fuel energy required for ignition. The remaining 70–80% inner energy can be provided by ps heating laser pulses via the high-current MeV electron beams guided by a ns-laser-produced magnetic field. The requirement for the heating energy from the ps heating laser pulses can be significantly reduced to an affordable level of the current PW laser technology.

The feasibility of the four progressive processes in the DCI scheme has been preliminarily examined by one-dimensional radiation hydrodynamic simulations and two-dimensional PIC simulations, respectively. It is shown that a quasi-isentropic compression with a density of approximately 150 g cm^−3^ and an areal density of 0.8 g cm^−2^ could be achieved with a 60 kJ total laser energy in the cone. By the acceleration and head-on collision processes, the high-density fuel could be preheated to a temperature approximately 1 keV with a doubled density. Finally, a fuel with an areal density of 0.8 g cm^−2^ could be ignited by a magnetic field-guided MeV electron beams produced by ps heating laser pulses with a total energy of 20–30 kJ.

In order to carry out the feasibility studies of the DCI scheme, a joint research team composed of experimental, lasers, diagnostics, target-fabrication and simulation groups has been formed from fifteen universities and four CAS institutes in China. At present, the Shenguang II laser facility is planned to perform an upgrading to provide an experimental platform to demonstrate the four processes progressively in next 5 years. Integrated experiments are planned to be implemented in 2024 and the proposed laser parameters are given in table 1.

## Data accessibility

This article has no additional data.
